# Familial aggregation of longevity in giant cell arteritis and polymyalgia rheumatica

**DOI:** 10.1007/s00296-020-04649-7

**Published:** 2020-07-18

**Authors:** Marcin Milchert, Marek Brzosko

**Affiliations:** Department of Rheumatology, Internal Medicine, Geriatrics and Clinical Immunology, Pomeranian Medical University in Szczecin, ul Unii Lubelskiej 1, 71-252 Szczecin, Poland

**Keywords:** Longevity, Mortality, Giant cell arteritis, Polymyalgia rheumatica

## Abstract

The long-term mortality in giant cell arteritis (GCA) and polymyalgia rheumatica (PMR) is unexpectedly decreased or at least not increased regardless of several mortality risk factors that these diseases share with other chronic immune-mediated rheumatic diseases. The genetic and immunological profile of PMR/GCA patients is unique, therefore, the hypothesis that this profile provides some survival advantage to PMR/GCA patients should be considered. The longevity is a phenomenon that was demonstrated to be familial. The familial aggregation of longevity can be studied by analysis of life expectancy in family members. Here we test the hypothesis of the aggregation of an increased longevity in the families of PMR/GCA patients. We compared the age of death of 358 parents of 179 PMR and GCA patients with corresponding data retrieved from 506 parents of 253 randomly collected age and sex-matched controls. The number of nonagenarian (≥ 90-year -old) mothers of PMR/GCA patients was significantly higher (OR = 2.34, 95%CI 1.11–11.95, *p* < 0.0005) vs controls. Both nonagenarian parents were found in 6 patients (3.35%) and none in the control cohort (OR = 8.77, 95%CI 2.26–405.10, *p* = 0.003). Our data suggest the familial aggregation of nonagenarians in PMR/GCA patients.

## Introduction

Giant cell arteritis (GCA) and polymyalgia rheumatica (PMR) are closely related, chronic immune-mediated rheumatic diseases, limited to elderly patients. The long-term mortality is at least not increased in GCA and PMR [1–5] or even decreased compared to the general population [6–9]. It is surprising because these diseases share several risk factors of increased mortality with other immune-mediated rheumatic diseases. The decrease in a lifespan in GCA was rarely observed [10, 11] or was minimal [12]. Inpatient GCA mortality was also reported to be decreased when compared to the matched inpatient population [13]. In addition, in isolated PMR (PMR without concomitant GCA) an increased survival was observed [2, 14].

The genetic background (high prevalence of these diseases in special populations, association with special HLA antigen) and immunological profiles (with strongly pronounced autoinflammatory component) of PMR/GCA patients’ are unique [6, 14, 15], therefore, we hypothesize that these might correlate with some survival advantage. Explanation of this potential advantage would imply a need for including some genetic or behavioural factors. Longevity is a phenomenon that was demonstrated to be familial, and is associated with the inherited longevity genes, the survival-promoting genes or health-promoting behaviours. The family members of long-lived subjects have a survival advantage. Familial aggregation of longevity can be studied by analysis of life expectancy in family members [16–18].

We focused on the unexpected survival advantage in PMR/GCA and tested the hypothesis of aggregation of an increased longevity in PMR/GCA patients and their families.

## Materials and methods

Information was retrieved from patients diagnosed in the single reference center for GCA/PMR between 2016 and 2019. We collected information from 179 consecutive PMR and GCA patients (aged 75.6 ± 8.7 years; min 52, max 97, 125 women, 54 men, 124 GCA, 129 PMR) and 253 controls (aged 76.4 ± 9.4 years; min 51, max 98, 153 women, 100 men). Age of death of 358 deceased parents of patients and 506 deceased parents of controls was noted. Age and sex-matched controls were randomly collected from inadvertently met people without the diagnosis of PMR/GCA, in the same time period and region (*N* = 112), recruited from patients’ spouses without the diagnosis of PMR/GCA (*N* = 42), and retrieved from open-access national polish genealogy database [19] (*N* = 99, the database enables collection of information on age and sex of current population group of people and their parent’s age of death, that were consecutively extracted). Nine patients were not included because of unawareness of their parents’ age of death.

The diagnosis of GCA/PMR was confirmed during at least one follow up visit within 1–6 months. GCA patients met 1990 ACR classification criteria [20] or presented GCA manifestations together with large vessel vasculitis confirmed by ultrasound, computed tomography and/or PET-CT. PMR patients met EULAR/ACR classification criteria [21] Isolated PMR presented no clinical manifestations of GCA and no temporal and axillary vasculitis that were excluded by ultrasound.

We compared the age of death of parents of PMR and/or GCA patients (including isolated PMR subgroup) with controls. The logistic regression and *t* Student’s test were applied due to a normal distribution that was checked by Kołmogorow–Smirnow test. The Spearman correlation coefficient was additionally used. *p* < 0.05 was considered significant. Statistical analyses were performed with STATA software (version 12.0; StataCorp).

The additional analysis was performed vs. the control group recruited from age and sex-matched patients (289 parents of 145 patients) referred with suspected PMR/GCA but diagnosed with rheumatoid arthritis (*N* = 37 patients), osteoarthritis (*N* = 23), other rheumatic conditions (*N* = 19), or no rheumatic disease (*N* = 39).

The study was accepted and patients’ consent was waived in accordance with the Pomeranian Medical University ethical committee decision KB-0012/170/06/19.

## Results

The number of nonagenarian (≥ 90-year-old) mothers of both female and male PMR/GCA patients was significantly higher in the following cohorts: GCA/PMR (Table 1), isolated PMR (without concomitant GCA, OR = 7.52, 95%CI 2.81–20.33, *p* < 0.00005), PMR (OR = 4.73, 95%CI 2.04–11.59, *p* < 0.00005), GCA (OR = 4.13, 95%CI 1.73–10.32, *p* = 0.0002), and isolated GCA (without concomitant PMR, OR = 6.08, 95%CI 2.10–17.28, *p* < 0.0000) compared to the population-based controls. No difference in the number of nonagenarian mothers between the isolated GCA vs. PMR (OR = 1.29, 95%CI 0.49–3.15, *p* = 0.5550) nor isolated PMR vs. GCA (OR = 1.82, 95%CI 0.75–4.33, *p* = 0.1368) was found. There were significantly fewer nonagenarian fathers compared to the controls (Table 1.), with a lower significance compared to an increased number in mothers (Table 1 and 2). Both nonagenarian parents were found in six patients (3.35%) and none in the controls. No significant difference in the mean length of life between PMR/GCA parents vs. controls was found (Table 3.).

**Table 1:** Number of nonagenarians (≥ 90-years-olds) in parents of PMR/GCA patients

	Parents age	PMR/GCA (*N* = 179)	Controls (*N* = 253)	OR	95%CI	*p*
Mothers	< 90	148 (82.68%)	243 (96.05%)			
	≥ 90	31 (17.32%)	10 (3.95%)	2.34	1.11–11.95	< 0.0005
Fathers	< 90 ≥ 90	161 (89.94%) 18 (10.06%)	203 (80.24%) 50 (19.76%)	0.45	0.24–0.83	0.0064
One of parents	< 90 ≥ 90	136 (75.98%) 43 (24.02%)	193 (76.28%) 60 (23.72%)	1.02	0.63–1.63	0.9412
Both parents	< 90 ≥ 90	173 (96.65%) 6 (3.35%)	253 (100.00%) 0 (0%)	8.77	2.26–405.10	0.003
Mothers of female patients	< 90 ≥ 90	105 (84%) 20 (16%)	149 (97%) 4 (3%)	7.1	2.27–29.19	0.0001
Mothers of male patients	< 90 ≥ 90	43 (80) 11 (20%)	94 (94%) 6 (6%)	4.01	1.25–13.97	0.0066

*GCA* giant cell arteritis, *PMR* polymyalgia rheumatica, *N* number of all parents

**Table 2:** Correlation between the number of nonagenarians (≥ 90 -years -olds) in parents in different PMR/GCA subsets

		*N*	Correlation coefficient	*p*
Number of nonagenarians in mothers	GCA	124	− 0.19	0.0002
	Isolated GCA (without concomitant PMR)	50	− 0,24	0.00002
	PMR	129	− 0.21	0.00003
	Isolated PMR (without concomitant GCA)	55	− 0.29	< 0.000005
Number of nonagenarians in fathers	GCA	124	0.16	0.0016
	Isolated GCA (without concomitant PMR)	50	0.07	0.1975
	PMR	129	0.13	0.0087
	Isolated PMR (without concomitant GCA)	55	0.03	0.5830

*GCA* giant cell arteritis, *PMR* polymyalgia rheumatica, *N* number of all parents

**Table 3:** Length of life of parents of PMR/GCA patients and controls

	Length of life ± SD (min–max)				
	PMR/GCA (*N* = 179)	GCA (*N* = 124)	Isolated PMR (without concomitant GCA) (*N* = 55)	Controls (*N* = 253)	*p*
All parents	74.6 ± 15.6	73.7 ± 15.6	74.6 ± 17.0	76.2 ± 11.3	> 0.05
Fathers	71.2 ± 15.9	70.1 ± 15.8 (30–100)	73.8 ± 16.1 (30–96)	79.8 ± 10.7 (38–99)	> 0.05
Mothers	77.9 ± 14.5	77.3 ± 14.5 (29–99)	79.2 ± 14.5 (30–97)	72.5 ± 10.8 (40–96)	> 0.05

*GCA* giant cell arteritis, *PMR* polymyalgia rheumatica, *N* number of all parents

The additional analysis vs control group recruited from the subjects referred with the suspected but excluded PMR/GCA revealed an increase in the number of nonagenarian parents of isolated PMR patients (OR = 2.10, 95%CI 1.11–3.94, *p* = 0.0121).

## Discussion

Previous studies have attempted to explain the phenomenon of increased life expectancy in PMR/GCA pointing to a closer medical surveillance of patients, i.e., better management of comorbidities, including cardiovascular disease [2, 13, 14]. However, such favourable effects were not reported in other immune-mediated rheumatic conditions, which in contrast have reduced life expectancy [6]. In addition, increased life expectancy in PMR/GCA patients is observed regardless of harmful effects of systemic inflammation, adverse effects of therapy [13], and a higher risk of dissecting aortic aneurysm development [12]. PMR/GCA patients seem not to be resistant to the disease-related risk factors because mortality within the first 2 years following diagnosis was reported to be increased suggesting cause-specific effect [1]. Therefore, we find it important to consider the additional factors that may favour survival in PMR/GCA patients.

An accepted model of studies of longevity encompass the analysis of environmental and genetic factors in nonagenarians (≥ 90 -years -olds) and centenarians (≥ 100 -years olds) [16, 17]. The family members of the long-lived subjects have a survival advantage. Longevity was demonstrated to be at least partially inherited in mitochondrial DNA [16, 17] which is transferred by mothers. This remains in line with our observation of an increase in the number of nonagenarian mothers of PMR/GCA patients. Interestingly, the combination of nonagenarian father and mother was highly more common in PMR/GCA compared to the controls. In previous studies, combined parental longevity was reported to be associated with a decreased all-cause mortality [18].

In addition, genetic regulation of longevity may be indirect, regulating susceptibility to age-related diseases i.e., cardiovascular disease [18]. This mechanism might be considered in GCA patients in whom cardiovascular profile was demonstrated unexpectedly favourable [22]. However, we were not able to compare cardiovascular disease and medical surveillance between our patients and controls to further investigate possible explanations for our finding. High intensity of innate immune activation in elderly patients with PMR/GCA is frequently surprising, questioning the process of downregulation of immune responses known as”immunoaging” in these patients. We speculate that unique immunologic profile (i.e., strong innate immunity responses) of PMR/GCA patients might potentially influence specific mortality causes. For example, it could impact life-threatening infections or neoplasms (their prevalence was discussed in PMR/GCA with conflicting results) [1]. Still, the previously favoured theory of “closer medical surveillance of PMR/GCA patients” favouring PMR/GCA patients survival cannot be excluded based on our observation. However, the decreased number of nonagenarian fathers of our patients remains unexplained. The correlation coefficient of decreased number of nonagenarian fathers was lower compared to increase in mothers and combination of nonagenarian parents. Studies in larger groups are required.

The survey method of data extraction may be considered a limitation of our study. However, only a few patients were unaware of their parents’ age at death. A strength of the study was that results were consistent in both isolated PMR and isolated GCA— both diseases share a genetic background. In addition, analysis in an additional control group recruited from patients with GCA mimics confirmed increased longevity of parents of patients with isolated PMR.

Previous data on unexpectedly low long-term mortality in PMR/GCA deserve attention. We found a familial aggregation of longevity in PMR/GCA patients by demonstrating an increased number of nonagenarian (≥ 90-year- old) mothers and both nonagenarian parents of PMR/GCA patients. Further studies warrant confirming our finding.
